# “I had no other choice but to catch it too”: the roles of family history and experiences with diabetes in illness representations

**DOI:** 10.1186/s12902-020-00580-x

**Published:** 2020-06-26

**Authors:** Amy T. Cunningham, Alexzandra T. Gentsch, Amanda M. B. Doty, Geoffrey Mills, Marianna LaNoue, Brendan G. Carr, Judd E. Hollander, Kristin L. Rising

**Affiliations:** 1 Department of Family and Community Medicine, Sidney Kimmel Medical College, Thomas Jefferson University, 1015 Walnut Street, Suite 401, Philadelphia, PA 401 USA; 2 Department of Emergency Medicine, Sidney Kimmel Medical College, Thomas Jefferson University, 1025 Walnut Street, Suite, Philadelphia, PA 300 USA; 3 Department of Emergency Medicine, Sidney Kimmel Medical College, Thomas Jefferson University, 1025 Walnut Street, Suite, Philadelphia, PA 300 USA; 4 Department of Family and Community Medicine, Sidney Kimmel Medical College, Thomas Jefferson University, 1015 Walnut Street, Suite, Philadelphia, PA 401 USA; 5 College of Population Health, Thomas Jefferson University, 901 Walnut Street, 10th floor, Philadelphia, PA USA

**Keywords:** Diabetes, Family history, Illness representations

## Abstract

**Background:**

A family history of diabetes and family members’ experiences with diabetes may influence individuals’ beliefs and expectations about their own diabetes. No qualitative studies have explored the relationship between family history and experiences and individuals’ diabetes illness representations.

**Methods:**

Secondary data analysis of 89 exploratory, semi-structured interviews with adults with type 1 or type 2 diabetes seeking care in an urban health system. Participants had a recent diabetes-related ED visit/hospitalization or hemoglobin A1c > 7.5%. Interviews were conducted until thematic saturation was achieved. Demographic data were collected via self-report and electronic medical record review. Interviews were audio-recorded, transcribed, and coded using a conventional content analysis approach. References to family history and family members’ experiences with diabetes were analyzed using selected domains of Leventhal’s Common Sense Model of Self-Regulation.

**Results:**

Participants cited both genetic and behavioral family history as a major cause of their diabetes. Stories of relatives’ diabetes complications and death figured prominently in their discussion of consequences; however, participants felt controllability over diabetes through diet, physical activity, and other self-care behaviors.

**Conclusions:**

Findings supported an important role of family diabetes history and experience in development of diabetes illness representations. Further research is needed to expand our understanding of the relationships between these perceptions, self-management behaviors, and outcomes. Family practice providers, diabetes educators and other team members should consider expanding assessment of current family structure and support to also include an exploration of family history with diabetes, including which family members had diabetes, their self-care behaviors, and their outcomes, and how this history fits into the patient’s illness representations.

## Background

Diabetes management is a shared decision-making process with collaborative goal setting and action planning regarding diet, physical activity, monitoring, medication management, healthy coping, and reducing risks [1]. Individuals’ diabetes treatment goals and self-management behaviors can be strongly influenced by their illness representations [2–4], also known as explanatory or mental models [5–7]. Illness representations are an individual’s beliefs about, and expectations of, their illness. Individuals construct their representations from a variety of sources, including their symptoms, self-care experiences, interactions with health care providers, and witnessing the experiences of family, friends, and others with an illness. Patient representations may differ significantly from those of diabetes educators, physicians, and other health professionals [8].

Leventhal’s Common Sense Model of Self-Regulation (CSM) is a widely-used framework for understanding how individuals construct illness representations in response to health threats such as diabetes or other chronic illnesses. According to the CSM, attributes of these representations are: 1) identity, e.g. naming of the condition/symptoms; 2) illness timeline, meaning its onset and perceived duration; 3) causes, such as genes, behavior, and the social environment; 4) consequences, both psychological and physical, and 5) controllability--whether the illness can be cured or controlled and what role the individual, medical professionals, and others play in controlling the illness. When a patient has a perceived health threat, these attributes shape their response, such as engagement in—or avoidance of--self-management behaviors [9, 10].

Family genetic history of disease is a known risk factor for development of both type 1 and type 2 diabetes. In addition, families often transmit dietary practices, physical activity patterns, and diabetes-related health beliefs and coping strategies across generations. Therefore, family members’ beliefs and experiences with diabetes can also be an important influence on an individual’s diabetes illness representation. In the following, “family history and experiences” will be used to encompass genetic, behavioral and environmental inheritance, and family members’ experiences living with diabetes.

Prior work has assessed perceptions of family history and experiences based on the CSM framework among individuals who have a family history but have not yet developed diabetes or other conditions, with the goal of developing effective risk-assessment and interventions to reduce or delay complications. Among these individuals, family history and experiences play an important role in an individual’s perceived illness cause, potential consequences, and perceived controllability of diabetes and other common diseases [11].

However, limited research exists on the impact of family history and experiences on illness representations among participants already diagnosed with diabetes. Some have proposed a “multigenerational legacy of diabetes” to describe the influences of a family history of diabetes, including observations of family members’ diabetes beliefs, self-care behaviors, and outcomes [12]. A survey examining the multigenerational legacy of diabetes among individuals with type 2 diabetes in a diabetes self-management education program found that those with a family history of diabetes reported higher levels of diabetes management self-efficacy and higher rates of alignment with dietary plans than those without a family history [13]. In contrast, another survey of adults with type 2 diabetes found that individuals with a family history of diabetes-related amputation, high levels of perceived risk of amputation, and high levels of fear reported lower levels of foot self-care than those without a family history of amputations [14].

These differing findings on the relationship between perceptions of family history and diabetes self-care suggests that family history can have complex and varied effects among individuals with diabetes, including their attitudes and approaches to self-management. To date, no qualitative studies have explored the influence of family history on diabetes illness representations, including the CSM domains, among those with diabetes. We report exploratory qualitative findings on the perceptions of diabetes family history and experiences on the illness representations of individuals with diabetes.

## Methods

### Research design

This study was a secondary analysis of semi-structured interviews comparing the comprehensiveness and efficiency of semi-structured interviews and group concept mapping for eliciting patient-important outcomes for diabetes care as part of a Patient Centered Outcomes Research Institute-funded methodology study. The full methods and findings of the primary study have been described elsewhere [15]. Only data from the semi-structured interviews are included in this secondary analysis.

### Setting

Interview participants were recruited from an urban academic medical center. Research staff recruited participants from three settings: 1) the emergency department (acute care), 2) within 7 days post-hospital discharge (post-acute care), and 3) scheduled primary care visits (primary care). Interviews were continued until thematic saturation was achieved for each setting, with a goal of 30 interviews per setting.

### Sample

The study sample was a convenience sample. Eligible individuals were English-speaking adults 18 and older with type 1 or type 2 diabetes mellitus who were able to provide written informed consent. All participants were required to have type 1 or type 2 diabetes mellitus (DM), who presented to the emergency department (ED) with a DM-related problem (acute care), admitted to the hospital for a DM-related problem (post-acute care), or at least 2 measurements of hemoglobin A1C (A1C) > 7.5 in the prior year (primary care). These individuals were chosen because study findings could be used to tailor care for this vulnerable population to reduce or delay future complications.

Exclusion criteria included participants with a new diagnosis of DM during that visit; having a significant permanent complication related to DM, including end stage renal disease, amputation, or blindness; undergoing medical clearance for a detox center or any involuntary court or magistrate order; in police custody or currently incarcerated; or having major communication barriers such as visual or hearing impairment or dementia that would compromise ability to give written informed consent.

Acute participants were screened using the ED’s electronic medical record (EMR) during scheduled shifts; research staff approached individuals as soon as they were identified as potentially eligible. For post-acute and primary care participants, research staff screened auto-generated lists from the inpatient and outpatient EMR to identify potential participants. Post-acute participants were contacted, consented and interviewed by telephone. Primary care participants were contacted by phone prior to a scheduled visit to provide information on the study and to assess interest in study participation. Participants were subsequently consented and interviewed on site immediately before or after their primary care appointment. All participants who were enrolled in-person provided written informed consent, and those enrolled over the telephone provided verbal consent. The informed consent process included description of the study’s purpose, the researcher’s role in the study, the study, funder, participant risks and benefits to participation, and assurance that refusal or noncompletion of the study would not affect their care. Interview participants were compensated $25. Research approval was obtained from Thomas Jefferson University’s institutional review board.

### Data collection

As part of the interview, participants completed demographics forms; last recorded A1C and body mass index (BMI) were obtained from the EMR. The authors used an open-ended, semi-structured interview guide to discuss outcomes most important to participants when making decisions regarding management of their DM. The guide was developed, tested, and refined in collaboration with our Patient and Key Stakeholder Advisory Board (PAKSAB). PAKSAB members included individuals with diabetes, caregivers for family members with diabetes and other chronic conditions, a diabetes educator,and a primary care physician Interview questions included participants’ belief about the causes of their diabetes and what it is doing to their body, worries and concerns about the illness, how well managed they considered their diabetes to be, their indicators of successful management, goals for their diabetes care, challenges caring for their diabetes, and anything else they wished to add about their diabetes care (Additional file 1).

Interviews were one-on-one, lasted approximately 30 min and were audio-recorded.. Interviews were completed by four study team members: two master’s prepared research coordinators with training in clinical research (ATG,AMBD) and two PAKSAB members, a patient advocate and a nurse practitioner, all female. All interviewers were trained and supervised by a senior researcher with extensive qualitative experience (KLR). All interviews were transcribed professionally with identifying information removed. Interviews were assigned identification numbers based on interview setting and sequence— “1” indicated interviews from the primary care setting, “2” from the emergency department setting, and “3” from post-hospital discharge. Transcripts were checked by a team member for accuracy.

### Data analyses

Interviews were analyzed using Vivo 11.0 software [16]. Interviews were coded by three team members, including two PAKSAB members. A fourth researcher performed double-coding of a portion of interviews to ensure coding consistency. A team member experienced in qualitative methodology oversaw the process. The coding team routinely reviewed the Kappa co-efficient and percentage agreement and coders regularly met as a group to address discrepancies. The minimum acceptable Kappa coefficient was set at 0.60, the target was 0.80, and the average coefficient was 0.70. For codebook development, the team decided a priori to develop a “goals” node (concept) to capture all ideas related to participant goals or treatment priorities, as this was the primary focus for the overall research project. Other nodes represented codes that emerged during the primary analysis process conducted using a conventional content analysis approach [17]. The coders developed the codebook by immersing themselves in the transcripts, identifying codes and subcodes that emerged, and coding independently. They then met to review coding, resolve discrepancies, and refine the codebook iteratively.

Information on family history was coded to the node “Family, friends, and others” and/or its “stories about others” subnode, which consisted of stories about family, friends and others’ diabetes experiences, including symptoms and complications. For the secondary analysis, content of these two nodes node and subnode was coded to the following three domains from the CSM framework: causes, consequences, and controllability [10] using directed content analysis. These domains were selected a priori based on the multigenerational legacy of diabetes literature, which has shown an association between these domains, family history, and self-management behaviors [12–14]. IBM SPSS Statistics 26 (IBM Corp., Armonk, N.Y., USA) was used to calculate descriptive statistics for participants and to conduct chi-square analyses of differences in family history themes by care setting (ED, post-acute care, and primary care).

### Rigor

Rigor was ensured through attention to study credibility, confirmability and dependability, and transferability [18]. Credibility was addressed by using data triangulation and member-checking. Triangulation was accomplished by interviewing participants from differing care settings (emergency department, primary care, and post-hospital discharge), through use of multiple coders, and assessment of intercoder reliability. Member-checking was provided by two PAKSAB members who served as coders, and the full PAKSAB reviewed and discussed interview findings with the research team. Upon conclusion of data analysis, summarized findings were disseminated to participants. Confirmability was supported through an audit trail. Detailed records were kept of all steps of the interview and coding process through a regularly-updated study procedures manual, meeting minutes, and retention of all copies of interview guides and coding trees. Research team members analyzed nodes’ contents and created narrative node summaries (memos) describing the nodes’ themes with links to relevant interview text. The team provided these study materials to PCORI along with biannual progress reports. The Consolidated Criteria for Reporting Qualitative Research (COREQ) checklist [19] was also used to ensure thorough reporting of the study procedures and findings. Transferability was supported through thorough description of the study participants and use of verbatim quotes to support themes.

## Results

A total of 126 individuals were approached for interviews: 31 declined, due primarily to lack of interest in participating or not feeling well enough to participate. Ninety-five were enrolled and 89 completed interviews; 2 dropped out of the study, 2 were ultimately ineligible because it was determined that they met exclusion criteria, and 2 recordings were not usable. The mean participant age was 55 years, with a standard deviation of 13 years. Sixty-eight percent of participants were Black, and the same percentage were high-school graduates. The mean A1C and standard deviation was 10.2% (3.3%) (13.7 mmol/l(4.4 mmol/l)). (See Table 1 for demographics). Interview content coded to the “family, friends, and others” node and “stories about others” subnode revealed a prominent role for family history and experiences in individual’s understanding of the causes of their diabetes, potential consequences, and its controllability. Family history themes did not vary significantly by care setting. *X*^*2*^(4, *N* = 89) =6.00, *p* = .199.

**Table 1:** Participant Demographics (*N* = 89)

Characteristic*	Mean (SD) or ***n***(%)
Age	54.6 (13.8)
Ethnicity	
Hispanic/Latino	8 (9)
Not Hispanic/Latino	80 (90)
Race	
White	24 (27)
Black	60 (68)
Other	4 (5)
Male	40 (45)
Female	49 (55)
A1C %	10.2 (3.3)
Mmol/l	13.7 (4.4)
Body Mass Index	34.8 (10.3)
Hospital Admits in past 12 months	2.3 (4.1)
ED Visits in past 12 months	2.8 (4.3)
Doctor Visits in past 12 months	11.2 (4.3)
Education	
Less than High School	4 (5)
High school graduate	68 (76)
College Degree	4 (5)
Post-Grad degree	13 (15)
Income	
< 10 K	15 (21)
10–25 K	22 (31)
25–50 K	19 (27)
50–99 K	7 (10)
> 100 K	8 (11)
Years since diagnosis	
< 1 year	2 (2)
1–5 years	12 (13)
> 5 years	74 (83)
Health status (mean, SD) (range 1–5,: 1 = excellent and 5 = poor)	3.6(0.9)

* All variables were self-report, aside from A1c and Body Mass Index

Note: Some percent totals do not equal 100, due to missing data

### Causes: perceived inevitability of diabetes due to family history

Many participants reported a history of family members with diabetes. Those with a family history of diabetes often expressed a sense of inevitability about developing the condition, saying that “It runs on my mother’s side and my father’s side, so I had no other choice but to catch it too” (ID 108). They felt that some of this inevitability was due to genetics, and would tally their relatives with diabetes: Often these lists spanned three or more generations and included grandparents, parents, aunts and uncles, and siblings: “My grandfather had diabetes, that would be my father’s father. And my grandmother had diabetes, that would be my father’s mother. My aunt on my father’s side – diabetes comes from their side. High blood pressure comes from my mom’s side. So that part, the genetic part, that’s the genetic part.” (ID 225). Another shared: I knew I was gonna have it because my whole family has it. I mean I remember my grandmother and she died in 1955. I remember my mother practicing on an orange with the real big syringes back then. It looked like you could drill for oil, you know, and giving her needles and all that… I think it’s just genes. I really do. Because for all of us kids to have it, and I’m the youngest of five, and every one of us has it (ID 311).

Although one participants attributed their diabetes solely to genetics, most thought it was a combination of genes and behavior, particularly eating habits: “I think it’s because of a couple of things. I never really learned the proper dietary. And also, my family has always been like that. My mother had it, my grandmother had it, my father had it, my grandfather had it. So it’s a long line that it not being taught correct diet and being overweight” (ID 326).Some reported eating patterns passed down through generations, such as family meals centered on fried foods and carbohydrates: “My mother and father, they was cooks. I mean, they cooked every day. They cooked fried chicken. I mean, they cooked everything going. Every day we had a hot cooked meal. We ate a lot of pork” (ID 112).

One participant felt that a combination of genes and dietary choices led to her diabetes: “I’ve always been a pasta, bread, sweets kind of girl. So having the predisposition with my father having it and his family having it, and then my mom’s side has had it, along with my eating habits just kind of set me up for it” (ID 110). Another reported having healthy food at home as a child, but said that her family history and later eating habits contributed to her diabetes:

But mainly it come from heredity and eating poorly too, even though my mom always did have good meals, vegetables and stuff. But as a kid, you don't look at that. Once you get 14, 15, you start basically buying your own food and eating big and eating out, and you just don't have no care in the world. But – and then heredity, it triggers it both so that I think that's how eating bad and having it in your bloodline, it just makes it worse (ID 124).

### Consequences: witnessing family members’ complications and deaths

When sharing their worries and concerns about their diabetes, many participants discussed family members’ diabetes-related complications and deaths, and expressed goals of avoiding similar complications and living long lives. Commonly-discussed complications of family members included amputations, particularly lower-limb amputations:

So this something that I – and it's good for me not to forget, but it's something I can't forget either because it's real and diabetes run in my family. And I have quite a few family members that passed from diabetes and had amputations from diabetes. It skipped my mother, but it got me. And so I'm very familiar with what – the dangers of it and what it can do to you if it's not managed (ID 217).

My mom had one leg cut off and they were getting ready to do her breast and she passed away. My older brother has all his toes on his left foot cut off and his right cut off in half, and I don’t wanna be like that. I don’t wanna feel pain. It’s bad enough I got pain from my neuropathy and nerve damage from my diabetes. I’m hoping after the holidays when I go get tested for heart disease that I don’t have it. Because my mom had heart disease too, which is bad. It’s fluid around the heart (ID 223).

Others discussed family members who had lost their eyesight

And another thing, people with diabetes, they always talk about numbers. What was your number? Mines was this, mines was that … That’s all – diabetes they talk about – probably because all the complications, that’s why. Because my husband, he had diabetes. His sugar was 900 and he went blind. So, that’s why – I see something in my eye right here – that’s why I came to the hospital. But he went blind and then he lost one leg and then he lost the other leg. And then he died. Scary. That’s about it, yeah” (ID 222).

A number also had relatives with end-stage renal disease who had to go on dialysis. Said one: “I actually lost my grandmother January 7th. And she was in the hospital about two months prior, and ended up on dialysis. She was a diabetic too and right afterwards, right after she started dialysis, she had a stroke and then she passed” (ID 129). Some worried that they would have the same fate: “My main concern – because my father died from end stage renal failure, that is one of my main concerns. My father, again – my father passed away from end stage renal failure. And my sister actually ended up – she had a kidney transplant” (ID 333).

Many attributed their family members’ declines to behavior: I’ve seen people who didn’t take care of their selves drinking, smoking, eating all kinds of things they shouldn’t be eating and not taking care of their self, losing limbs, going blind” (ID 111). One said of her mother: “She had diabetes. She found out when she was 30. She’s 68, about to be 69. But when I look at her, she’s deteriorating. She had a heart and kidney transplant. Her sight is gone. Her legs, she can barely walk. But she – it’s like the older generation, they don’t care” (ID 124).

Participants described progression of the disease among their family members through complications and deaths: examples included amputations, then dialysis, then death, or dialysis followed by a fatal stroke. A number also had family members die at young ages from diabetes: one individual said of her brother, “he went through the amputation and he wind up on kidney dialysis. And then he passed away at the age of 33 because of kidney failure” (ID 119). Another shared, “Well, I have – my kids, I worry about them losing their dad. Because my mom had diabetes and she only lived to 62 …My mom had to get dialysis. And yeah. I don’t want that to happen to me” (ID 227).

Witnessing family members’ physical complications—and in some cases, premature deaths—was a major driver behind participants’ goals of avoiding complications and living longer. One shared, “I had a couple family members that died from cancer and diabetes, then I started taking diabetes more serious. And then I started getting into studies about diabetes, what it can do to you, how you can lose your limb, how you can lose your eyesight. And I – it just – it bothers me” (ID 226). For some, their family members’ experiences resonated more so than advice from health professionals: I’ve talked to my nurse and I’ve talked to my doctors and I’ve talked to my dietician and noneof them really had a real deep, how do you say, impact on me. Like I said, my mother had diabetes and my grandmother had diabetes and none of them had taken it serious in their earlyyears. And it was not until I started seeing my mother – I don’t think that she understood that she was taking it serious but because someone else was taking care of her and was managing her food intake and was managing her medicine, that’s when I started thinking serious. My doctor told me how serious it was but I just didn’t listen (ID 310).

### Controllability:“We Are More Aware of It”

While many participants reported family members with diabetes experiencing multiple negative illness-related consequences, they did not all think that it was inevitable that their diabetes would progress the same way. Some described witnessing family members’ diabetes-related complications and deaths as powerful motivation for behavior change. They felt that they possessed more knowledge of diabetes and the consequences of self-care than prior generations—one described his aunt’s amputations as “old stupid stuff”:

You got to use common sense with this thing because see, my mother got it. My two aunts had it. My sister had it. And it killed all three of them, all except my mother. My mother's 93 years old. She's still here by using common sense. So, hey, follow mom's thing. Hey, I'm doing good. And I done had – first time I noticed my aunt was in 1956. She had diabetes, and they had to take both her legs – old stupid stuff – stuff that could be avoided today. You gotta know what's the time of day it is. (ID 102).

Individuals described several strategies to manage their diabetes through medical monitoring, diet, physical activity, and medications. For example, one participant’s mother and brother both had amputations, which she attributed in part to her eating habits; this made her set a goal to improve her diet. Another noted family members’ lack of physical activity as a contributor to their health and diabetes complications. As a result, he made a conscious effort to walk as a way of controlling his diabetes:

Not going blind or having my legs cut off. That’s the – honestly, that’s the most important thing. That’s why I catch the train to work because I can get off at [street] and I have to walk for about 15 to 20 minutes to get to work. And I know that that’s a big deal. And I had relatives that will get in the car and go around the corner to the corner store. So I just try to avoid that. I don’t work out constantly or do any of that stuff. So I try to do whatever little stuff I can do to make sure nothing bad happens to me (ID 201).

Several participants said they seek medical care more than their relatives, some of whom were unaware of their diabetes: one stated that his brother had not known he had diabetes prior to having a stroke—unlike his brother, the participant had been diagnosed with diabetes and received treatment early on, which he credited as the reason for his better health:

There are so many members of my family that have diabetes – all of my sisters. I had another brother that passed away. He was about 56 when he passed away in 2002. And he was a heavy drinking. And he did not take the diabetes medicine at all. And he took a heart attack and passed away. Yeah. Way young, and he was a diabetic. So there’s a lot of people in my family that’s diabetic. I have another sister. She’s ill. And my brother that passed away last year, he had a major stroke when he passed away. But he had another stroke before that. My sister had a stroke last year. And that’s why I’m trying to change my eating habits and whatnot, lose some weight. And weight is a factor, too (ID 206).

One participant described losing her mother to a diabetes-related coma, which she attributed to her mother not taking medications properly. This drove her to follow her own medication regimen carefully: “By the time my niece came back with her insulin and her medicine, she was in a coma and she never responded, never woke up … And of course, that’s always in the back of your mind. That’s what make me take– know I have to take my medicine. It runs in your family. You don’t want to go out like your mother did” (ID 112).

Most individuals cited relatives’ diabetes experiences as things to avoid, rather than successful management stories. However, a few cited examples of family members who were successfully managing their diabetes—one shared, “My grandpop who had diabetes, he lived to be 87 years old, and he didn’t have to be in a wheelchair until he was 85. So if I can get somewhere around there, that’s cool. He took care of himself” (ID 201).

## Discussion

Our study provides the first qualitative data on the impact of the perceptions of family diabetes history and experiences on diabetes illness representations. The current study also expands the literature on illness representations of diabetes; a recent scoping review of studies exploring type 2 diabetes illness representations, as defined by Leventhal’s CSM, reported no studies that examined the impact of family history and experience with diabetes [20]. Our findings showed that a family history of diabetes diagnosis and family members’ experiences with the illness play an important role in individuals’ illness representations, specifically for the attributes of causes, consequences, and controllability, also providing further support for the multigenerational legacy of diabetes theory [5–7].

Family history, both genetic and environmental, is seen as a major cause of diabetes, and a number of participants had multiple affected relatives, thus impacting their assessment of causality. Many family members had negative consequences such as complications and early death, influencing the assessment of consequences, As a result, avoiding complications and living longer were popular goals for participants. Notably, most examples of family members were of perceived lack of self-management and undesirable outcomes. Finally, regarding controllability, despite the lack of positive diabetes self-management experiences in their family, interviewees described feeling some influence over the illness through avenues such as diet, exercise, and medication.

Our findings regarding diabetes causality, consequences and controllability are similar to those for individuals without diabetes or other chronic illnesses who have a family history of the disease state. A qualitative meta-ethnography found that family history plays an important role in their risk perceptions: individuals developed a personalized sense of vulnerability based on the number of affected relatives, the emotional and physical closeness of that relative, and that relative’s illness experience. Despite feeling vulnerable, they felt that they had some control over their risk of developing the disease [11]. These findings, along with those from the current study, suggest that witnessing family members’ diabetes experiences could serve as a motivator for goal-setting and carrying out risk-reduction behaviors.

Our study does also have limitations: our sample was an urban, primarily Black population with recent diabetes-related ED visits/hospital admissions or elevated A1c, which may limit the transferability of findings to other settings and populations. While we randomly selected individuals to approach from among those eligible, we were unable to contact some individuals and others declined to participate, which may contribute to selection bias. Additionally, family was a theme that emerged from our findings, rather than an a priori focus. Therefore, future qualitative studies could delve deeper into the role of family history and prior experience in diabetes illness representations. Finally, as an exploratory qualitative study, we cannot draw conclusions about associations between perceptions of family history, self-care behaviors, and clinical outcomes. However, qualitative data is valuable in generating hypotheses which could be explored in future quantitative studies. Strengths of the study include its recruitment of individuals with diabetes from multiple care settings (acute, post-acute, and primary care) and the large sample, which reflected performing interviews to saturation in all settings. Our patient advisory board’s involvement in all phases of the project also enhances the credibility of the findings.

## Conclusions

Family history of diabetes and family members’ experiences with diabetes may shape participants’ illness representations, specifically regarding perceptions about the causes and consequences of diabetes and the controllability of the illness. Current American Diabetes Association guidelines for diabetes self-management education standards recommend that discussion of the individual’s current family structure and social support [1], but do not mention diabetes family history and experiences. As part of individualized education, physicians, diabetes educators, and other primary care team members would benefit from exploring family history and experiences, including which family members had diabetes, their self-care behaviors, and their outcomes, and how this history fits into the patient’s understanding of diabetes causes, consequences and controllability.

For some, fear of experiencing similar outcomes as their family members may lead to avoidant behaviors; for others, a desire to avoid complications or other negative outcomes could serve as a motivator to engage in self-care behaviors. If the patient expresses fear or grief regarding their family history of diabetes and its implications for their self-management and illness trajectory, that presents an opportunity to correct potential misperceptions of their risk of certain outcomes, discuss healthy coping strategies, and to engage in problem-solving to develop other self-management strategies [12]. Finally, those without family role models of successful diabetes self-management could benefit from group diabetes education or other forms of peer support.

## Data Availability

The datasets used and/or analyzed during the current study are available from the corresponding author on reasonable request.

## Abbreviations

A1C: Hemoglobin A1C
COREQ: Consolidated criteria for reporting qualitative research
CSM: Leventhal’s common sense model of illness regulation
DM: Diabetes mellitus
ED: Emergency department
EMR: Electronic medical record
PAKSAB: Patient and key stakeholder advisory board
